# Supplementary material for: Major urinary protein (Mup) gene family deletion drives sex-specific alterations in the house-mouse gut microbiota
Source: Microbiol Spectr. 2024 Jan 3;12(2):e03566-23. doi: 10.1128/spectrum.03566-23 (PMC10846032; doi:10.1128/spectrum.03566-23)
Supplement: Supplemental material — Supplemental results; Figures S1 to S6; Tables S1 to S8. [file spectrum.03566-23-s0001.pdf]

Supplemental Materials for Real et al., 2023

1. Supplemental Results
2. Supplemental Figures (Figure S1 – S6)
3. Supplemental Tables (Table S1 – S8)

1. Supplemental Results

To investigate the effect of sampling bias on the detection of significant effects of *Mup* deletion on the microbiota in males but not in females, we subsampled our WT males by alternatively removing one male from the analyzes and testing for differences in microbiota beta and alpha diversity and differential abundance at both taxonomic and functional (COG) profiles. PERMANOVA analyses testing for differences in species composition in the gut microbiota of WT and KO males revealed that significance was maintained in 3/4 cases based on Jaccard dissimilarities, and in 1/4 cases using Bray-Curtis dissimilarities (Table S7). In terms of COG-function composition, significance was maintained in 2/4 subsampling analyses based on Bray-Curtis dissimilarities but not maintained for analyses based on Jaccard dissimilarities (Table S7). Significant differences in microbial family Shannon diversity were observed for 1/4 cases (Table S8), but not for alpha diversity measures based on COG functions (Table S8). Importantly, subsampling had no qualitative effect on the detection of differentially abundant taxa or functions in males. The significant effects of *Mup* deletion in males observed in these subsampling analyses—particularly the male-specific differential taxon and COG-function results, which were completely robust to subsampling—lend further support to our conclusions that *Mup* deletion more strongly affected the microbiota of male mice than female mice.

2. Supplemental Figures

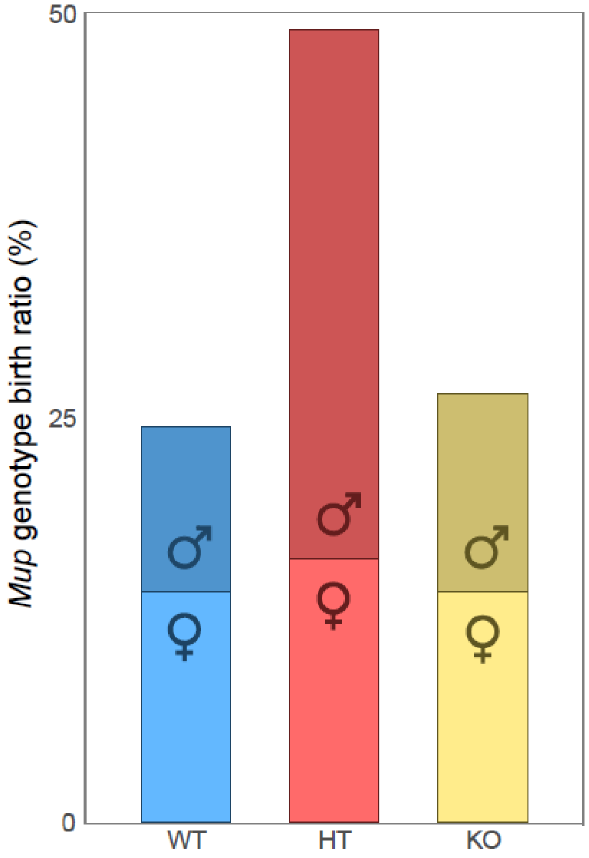

Figure S1. *Mup* genotype birth ratio. Bar plot shows the total percentage of male (top bar) and female (bottom bar) wildtype (blue), heterozygote (red), and knockout (yellow) pups born in the six study litters.

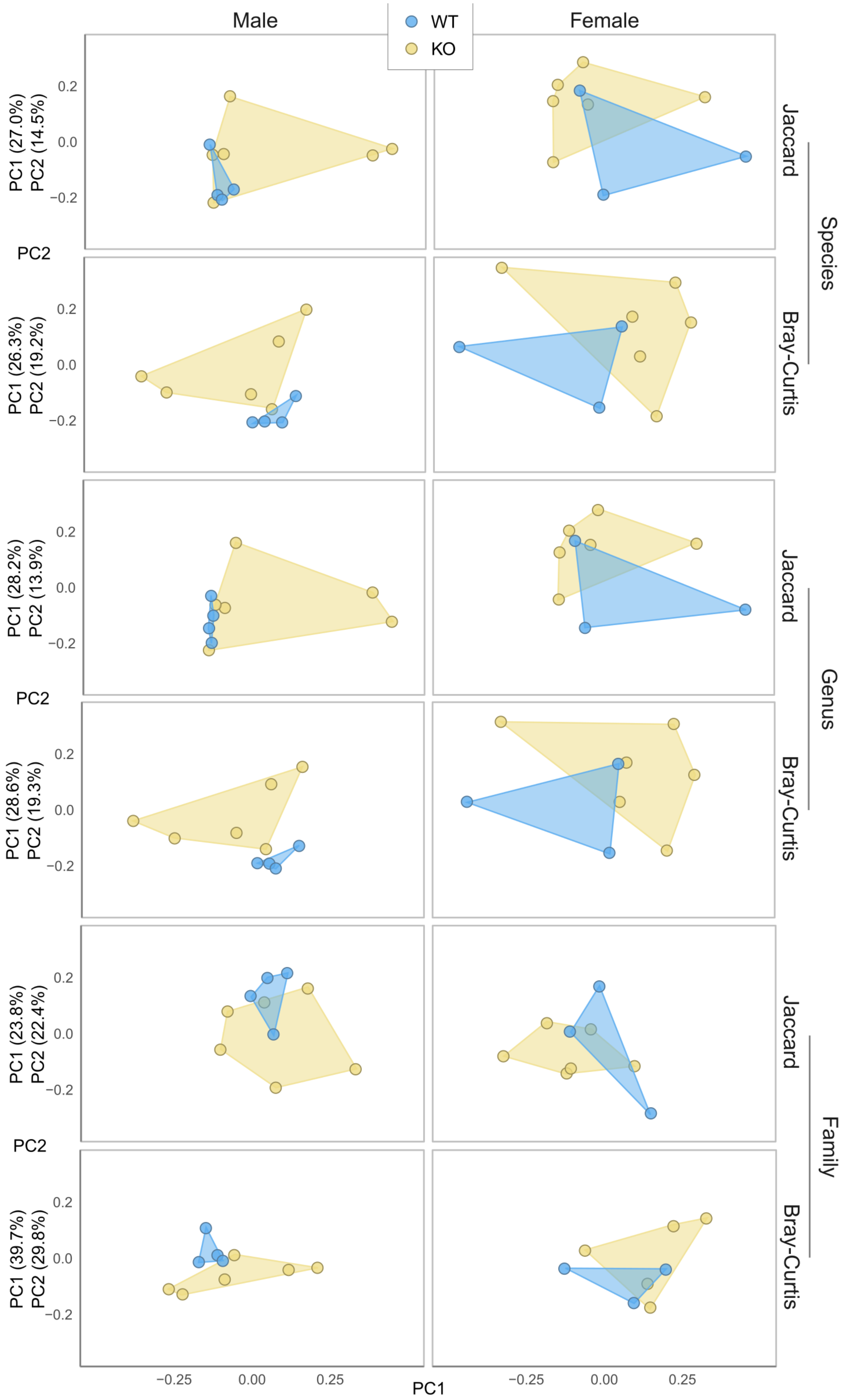
Male
Female
WT
KO
PC1 (27.0%)
PC2 (14.5%)
PC1 (26.3%)
PC2 (19.2%)
PC1 (28.2%)
PC2 (13.9%)
PC1 (28.6%)
PC2 (19.3%)
PC1 (23.8%)
PC2 (22.4%)
PC1 (39.7%)
PC2 (29.8%)
PC2
0.2
0.0
−0.2
−0.25
0.00
0.25
PC1
Jaccard
Bray-Curtis
Species
Genus
Family

Figure S2. *Mup* deletion significantly changes the gut microbial taxonomic composition of mature males. Principal coordinates analyses (PCoA) show the ordinated Jaccard and Bray-Curtis dissimilarities in Species, Genus, and Family composition in the fecal metagenome of sexually mature mice. The ordinated points are faceted by sex and colored by genotype, showing the taxonomic profiles of WT (blue) and KO (yellow) male (left column) and female (right column) mice. The percentage of variation in the taxonomic dissimilarity matrix explained by the first two PCo axes is enclosed in parenthesis.

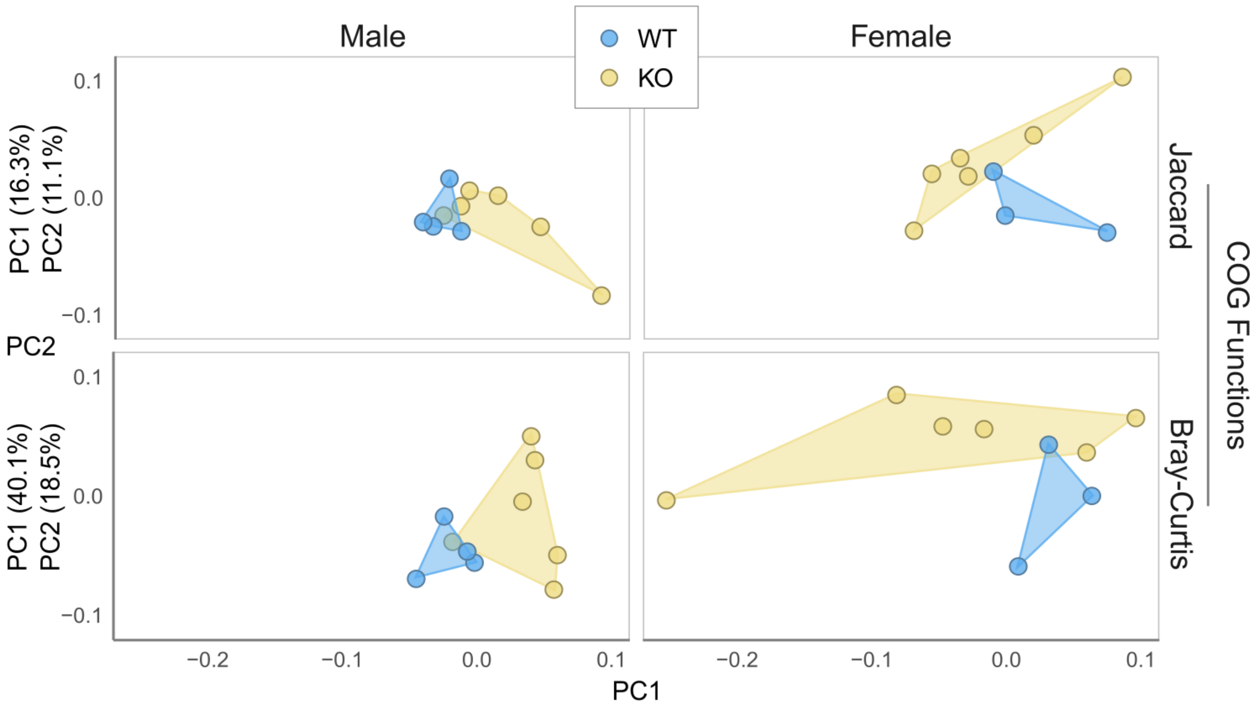

Figure S3. *Mup* deletion significantly changes the gut microbial functional composition of mature males. Principal coordinates analyses (PCoA) show the ordinated Jaccard and Bray-Curtis dissimilarities in COG Functions composition in the fecal metagenome of sexually mature mice. The ordinated points are faceted by sex and colored by genotype, showing the functional profiles of WT (blue) and KO (yellow) male (left column) and female (right column) mice. The percentage of variation in the taxonomic dissimilarity matrix explained by the first two PCo axes is enclosed in parenthesis.

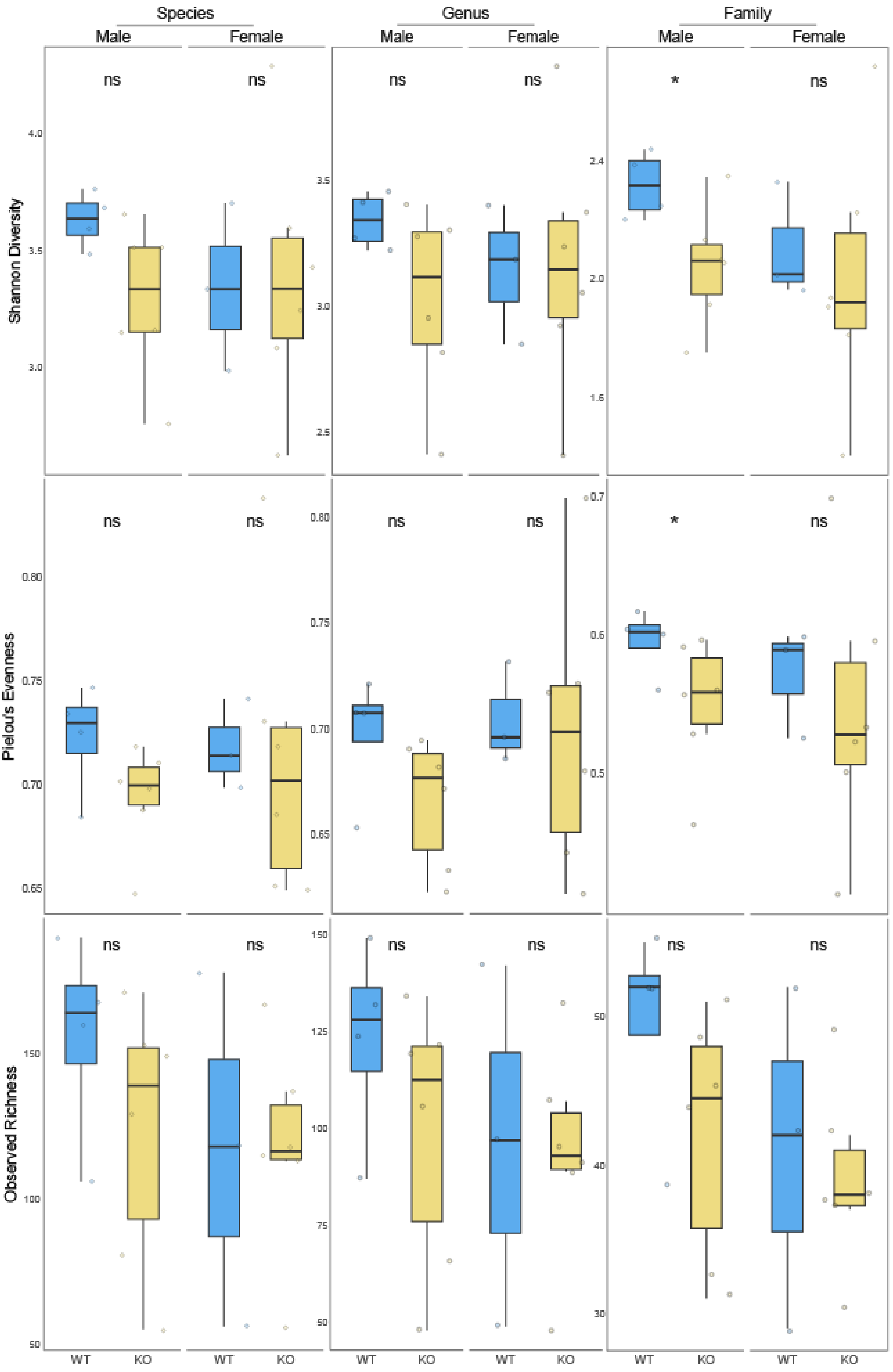
Species
Genus
Family
Male
Female
Shannon Diversity
Pielou's Evenness
Observed Richness
ns
*
WT
KO

Figure S4. *Mup* deletion significantly reduces microbial family diversity in mature males. Box plots show the Shannon Diversity (top row), Pielou's Evenness (middle row), and Observed Richness (bottom row) in microbial species (left column), genus (middle column), and families (right column) in the gut microbiota of mature males (left sub-column) and females (right sub-column). The linear mixed-effects model analyses (center top) indicate whether there is a significant difference in diversity in the gut microbiota of the WT (blue) and KO (yellow) mice (* = p-value < 0.05; ns = p-value > 0.05).

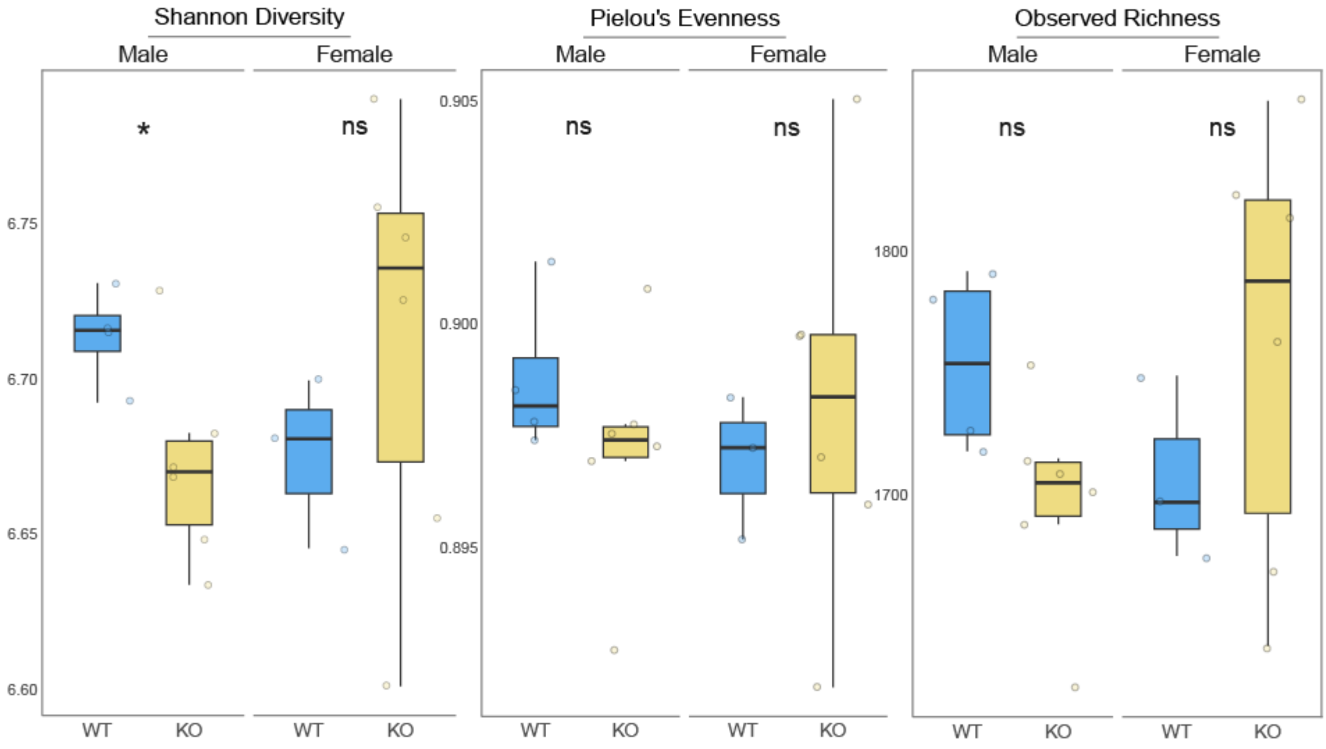

Figure S5. *Mup* deletion significantly reduces microbial functional diversity in mature males. Box plots show the Shannon Diversity (left column), Pielou's Evenness (middle column), and Observed Richness (right column) in microbial COG Functions in the gut microbiota of mature males (left sub-column) and females (right sub-column). The linear mixed-effects model analyses (center top) indicate whether there is a significant difference in diversity in the gut microbiota of the WT (blue) and KO (yellow) mice (* = p-value < 0.05; ns = p-value > 0.05).

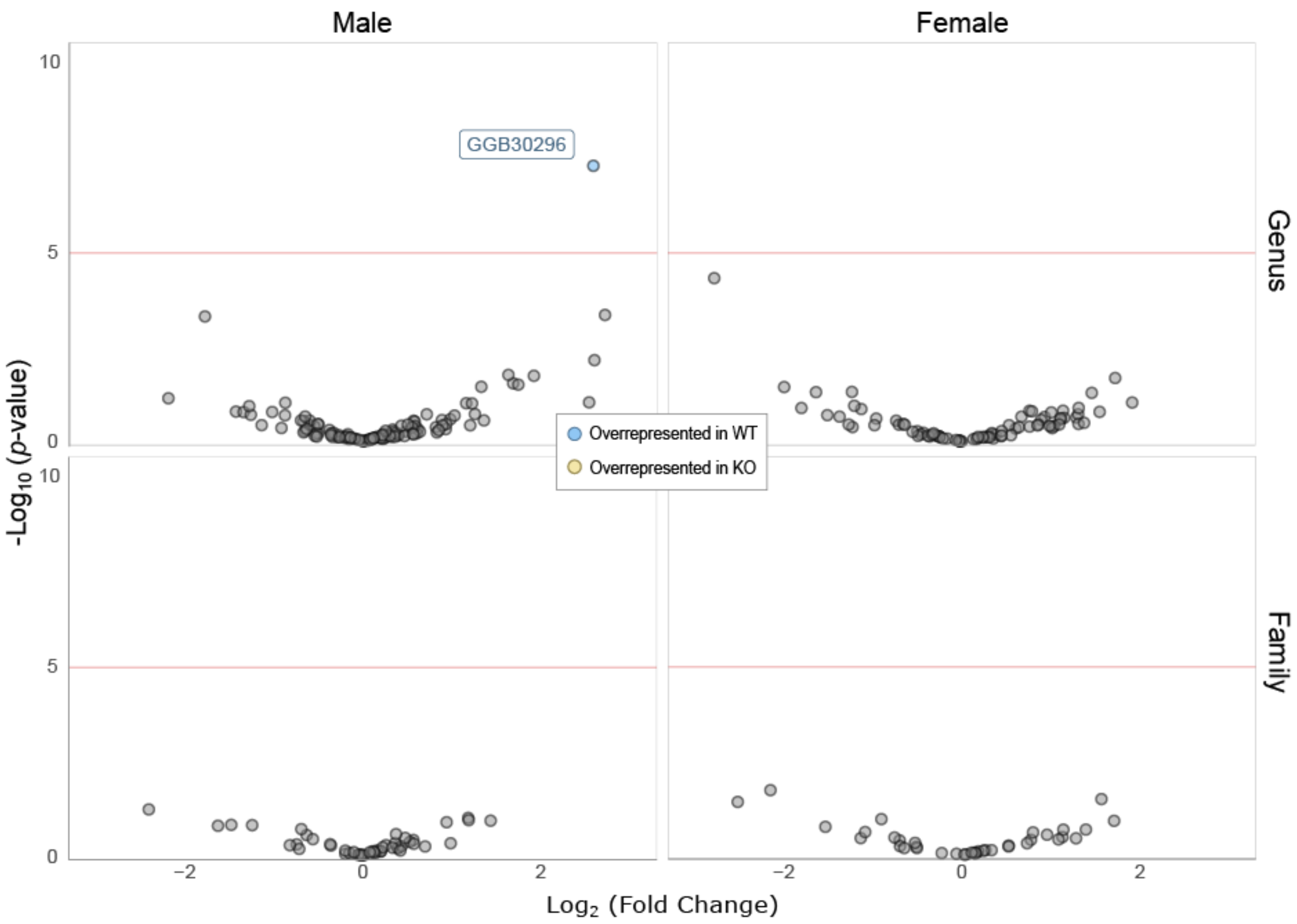

Figure S6. *Mup* deletion significantly shifts the abundance of various microbial taxa. Volcano plots show the log_2-transformed fold change (LFC) in the abundance of genus (top row) and families (bottom row) in the gut microbiota of mature male (left column) and female (right column) mice. ANCOM-BC2 analyses identified taxa (points) that were significantly more abundant in WT (blue) or KO mice (yellow). Red lines mark the significance threshold (Holm–Bonferroni–adjusted p-value < 0.001). The y-axis indicates the $-log_{10}$ transformation of the non-adjusted p-value.

3. Supplemental Tables

Table S1. Effect of host sex on microbial taxonomic and functional composition (*p*-value).

		WT		KO	
**Rank**	**Dissimilarity**	PERMANOVA	PERMDISP	PERMANOVA	PERMDISP
**Species**	Jaccard	**0.030**	0.324	0.057	0.900
	Bray-Curtis	0.061	0.284	**0.025**	0.423
**Genus**	Jaccard	**0.019**	0.291	**0.014**	0.840
	Bray-Curtis	0.087	0.282	**0.007**	0.385
**Family**	Jaccard	0.062	0.675	**0.031**	0.557
	Bray-Curtis	**0.035**	0.806	**0.047**	0.441
**COG Function**	Jaccard	0.089	0.282	**0.009**	0.969
	Bray-Curtis	**0.050**	0.188	0.057	0.090

Table S2. Effect of *Mup* genotype on microbial taxonomic and functional composition (*p*-value).

		Male		Female	
**Rank**	**Dissimilarity**	PERMANOVA	PERMDISP	PERMANOVA	PERMDISP
**Species**	Jaccard	**0.037**	0.273	0.154	0.739
	Bray-Curtis	**0.019**	0.188	0.158	0.398
**Genus**	Jaccard	**0.025**	0.175	0.171	0.935
	Bray-Curtis	**0.009**	0.333	0.146	0.397
**Family**	Jaccard	0.197	0.174	0.191	0.947
	Bray-Curtis	0.061	0.126	0.404	0.169
**COG Function**	Jaccard	**0.021**	**0.046**	0.116	0.633
	Bray-Curtis	**0.012**	0.080	0.481	0.200

Table S3. Microbial taxonomic and functional profiles correspondence with Procrustes (*p*-value).

Rank	Dissimilarity	Male	Female
**Species + COG**	Jaccard	**0.002**	0.953
	Bray-Curtis	**0.001**	0.230
**Genus + COG**	Jaccard	**0.001**	0.943
	Bray-Curtis	**0.001**	0.221
**Family + COG**	Jaccard	**0.001**	0.866
	Bray-Curtis	**0.001**	0.920

Table S4. Effect of *Mup* genotype on microbial taxonomic and functional diversity (*p*-value).

		**Male**		**Female**	
**Rank**	**Metric**	*p*-value	Estimate	*p*-value	Estimate
**Species**	Shannon	0.208	0.130	0.980	0.007
	Richness	0.320	8.534	0.844	-4.424
	Evenness	0.330	0.016	0.609	0.018
**Genus**	Shannon	0.371	0.092	0.895	0.036
	Richness	0.356	3.495	0.933	0.745
	Evenness	0.428	0.019	0.693	0.017
**Family**	Shannon	**0.041**	0.242	0.537	0.164
	Richness	0.481	-1.390	0.968	-0.093
	Evenness	**0.041**	0.078	0.455	0.052
**COG Function**	Shannon	**0.050**	0.041	0.484	-0.032
	Richness	0.594	10.490	0.476	-41.561
	Evenness	0.314	0.002	0.642	-0.001

Table S5. Microbial taxa and functions showing differential abundance in WT vs. KO genotypes*

Rank	Sex	Taxa / COG Function	*p*-value	LFC	Overabundant in:
**Species**	Male	SGB43260	**1.078E-05**	2.595	WT
	Female	SGB41239	4.657E-03	-2.784	KO
**Genus**	Male	GGB30296	**6.324E-06**	2.584	WT
	Female	GGB28621	4.286E-03	-2.781	KO
**COG Function**	Male	Na+/pantothenate symporter	**2.874E-05**	2.796	WT
	Male	Uncharacterized protein, possibly involved in motility	1.129E-03	1.667	WT
	Male	Predicted hydrocarbon binding protein (contains V4R domain)	1.042E-02	1.434	WT
	Male	L-asparaginase II	**1.534E-08**	1.427	WT
	Male	Trans-aconitate methyltransferase	2.487E-02	1.427	WT
	Male	Cu/Zn superoxide dismutase	**1.102E-04**	1.390	WT
	Male	V8-like Glu-specific endopeptidase	**1.475E-04**	1.385	WT
	Male	Type III secretory pathway, component EscU	**2.440E-09**	1.308	WT
	Male	Fucose 4-O-acetylase and related acetyltransferases	**4.605E-06**	1.201	WT
	Male	Cephalosporin hydroxylase	**5.297E-06**	1.104	WT
	Male	Uncharacterized protein required for formate dehydrogenase activity	**1.398E-05**	1.101	WT
	Male	Sugar diacid utilization regulator	3.722E-02	1.095	WT
	Male	Sulfite oxidase and related enzymes	1.690E-02	0.989	WT
	Male	Precorrin-2 methylase	3.460E-03	0.850	WT
	Male	Distinct helicase family with a unique C-terminal domain including a metal-binding cysteine cluster	1.379E-03	0.833	WT

	Male	Dissimilatory sulfite reductase (desulfoviridin), alpha and beta subunits	3.145E-02	0.788	WT
	Male	Uncharacterized protein conserved in cyanobacteria	**3.573E-04**	0.725	WT
	Male	Predicted transcriptional regulators containing the CopG/Arc/MetJ DNA-binding domain	**1.039E-04**	-0.819	KO
	Female	Glycosylphosphatidylinositol transamidase (GPIT), subunit GPI8	3.456E-03	2.142	WT
	Female	Uroporphyrinogen-III synthase	7.652E-03	-1.781	KO
	Female	Arginine kinase	2.754E-03	-1.915	KO

* Highly significant results are **bolded** (Holm–Bonferroni adjusted p-value < 0.001).

Table S6. Functional enrichment analyses with a hypergeometric test (*p*-value).

	**Male**		**Female**	
**COG Category**	**WT**	**KO**	**WT**	**KO**
Cellular processes and Signaling	0.545	1.000	0.205	1.000
Information storage and Processing	1.000	0.170	1.000	1.000
Metabolism	0.425	1.000	1.000	0.189
Poorly characterized	0.251	1.000	1.000	1.000

Table S7. Power analysis of taxonomic and functional beta diversity results (*p*-value).

Rank	Dissimilarity	w/o 219	w/o 224	w/o 256	w/o 257
**Species**	Jaccard	**0.014**	**0.039**	0.058	**0.041**
	Bray-Curtis	0.119	0.069	0.153	**0.046**
**Genus**	Jaccard	0.052	0.549	0.118	0.097
	Bray-Curtis	**0.017**	0.097	0.077	**0.029**
**Family**	Jaccard	0.320	0.138	0.100	0.431
	Bray-Curtis	0.084	0.126	0.053	0.175
**COG Function**	Jaccard	0.051	0.089	0.108	0.171
	Bray-Curtis	**0.032**	**0.018**	0.052	0.120

Table S8. Power analysis of taxonomic and functional alpha diversity results (*p*-value).

Rank	Metric	w/o 219	w/o 224	w/o 256	w/o 257
**Species**	Shannon	0.209	0.806	0.567	0.565
	Richness	0.613	0.053	0.685	0.677
	Evenness	0.259	0.102	**0.024**	**0.034**
**Genus**	Shannon	0.331	0.567	0.859	0.868
	Richness	0.795	**0.050**	0.522	0.516
	Evenness	0.252	0.138	**0.025**	**0.027**
**Family**	Shannon	**0.034**	0.184	0.077	0.117
	Richness	0.185	0.084	0.629	0.494
	Evenness	0.145	0.229	0.095	0.133
**COG Function**	Shannon	0.087	0.057	0.102	0.152
	Richness	0.373	0.559	0.558	0.546
	Evenness	0.802	0.141	0.303	0.399
